# Community-based identification of poor sleep quality in middle-aged and older adults with abdominal obesity using psychological and physical fitness indicators

**DOI:** 10.3389/fpubh.2026.1964356

**Published:** 2026-10-21

**Authors:** Chuyuan Qiao, Shenglei Yang, Yan Wang, Chuwei Yang, Yinghui He, Shuyi Zeng

**Affiliations:** School of Sports Medicine and Rehabilitation, Beijing Sport University, Beijing, China

**Keywords:** abdominal obesity, anxiety, depression, healthy aging, physical fitness, sleep quality

## Abstract

**Background:**

Poor sleep quality, abdominal obesity, psychological symptoms, and declining physical function may cluster during midlife and later life, posing a multidimensional challenge to healthy aging. We aimed to develop and internally validate a community-oriented nomogram integrating readily obtainable psychological and physical fitness indicators to identify current poor sleep quality in community-dwelling middle-aged and older adults with abdominal obesity and to evaluate its added value over routinely available demographic and health information.

**Methods:**

This retrospective cross-sectional study analyzed data from 212 adults aged ≥45 years with abdominal obesity assessed at a community health service center in Beijing, China. Poor sleep quality was defined as a Pittsburgh Sleep Quality Index (PSQI) score >5. Ten candidate predictors were evaluated using least absolute shrinkage and selection operator logistic regression with repeated stratified 10-fold cross-validation, followed by multivariable logistic regression. Model performance was assessed using the area under the receiver operating characteristic curve (AUC), calibration measures, the Brier score, and decision curve analysis. Separate 1,000-resample bootstrap procedures assessed variable-selection stability and optimism in the performance of the fixed seven-predictor model.

**Results:**

Among the 212 participants, 117 (55.2%) had poor sleep quality. The final model included age, the 9-item Patient Health Questionnaire, the 7-item Generalized Anxiety Disorder scale, grip strength, 30-s chair stand repetitions, chair sit-and-reach distance, and 2-min step count. The apparent AUC was 0.779 [95% confidence interval (CI), 0.718–0.840]. After bootstrap optimism correction, the AUC, calibration slope, and Brier score were 0.751, 0.858, and 0.206, respectively. Compared with the routine-information model (apparent AUC, 0.648), the final model improved the AUC by 0.131 (95% CI, 0.050–0.213; *p* = 0.002) and showed greater theoretical net benefit across threshold probabilities of 13–80% in the development sample.

**Conclusion:**

The nomogram showed moderate internal performance and higher apparent performance than the routine-information model, although content overlap between PHQ-9 and PSQI may have inflated these estimates. Where psychological questionnaires and field-based fitness assessments are already available, it may support targeted sleep assessment in community healthy-aging programs. External validation is required before implementation in public health practice.

## 1 Introduction

Poor sleep quality is common among older adults. A systematic review and meta-analysis of 995,544 community-dwelling older adults from 36 countries estimated a pooled prevalence of approximately 40% (1). Middle-aged and older adults with poor sleep quality are more likely to have concurrent depression, anxiety, functional limitations, reduced quality of life, and a greater burden of chronic disease (2–5). Sleep disturbances are also associated with obesity, particularly abdominal obesity. Short sleep duration has been associated with central obesity in adults and obesity in older adults (6, 7), and an association between poor sleep quality and abdominal obesity has also been reported in Chinese populations (8). Abdominal obesity is clinically used as a marker of central adiposity and may reflect the burden of metabolically active visceral fat. Visceral adipose tissue has been associated with systemic inflammation and oxidative stress beyond conventional measures of overall adiposity (9). Experimental sleep restriction may also alter leptin, sympathovagal, and glucoregulatory profiles (10) and impair insulin signaling in adipocytes (11). These findings provide a plausible endocrine–metabolic context for the close relationship between disturbed sleep and abdominal obesity. Among older adults with obesity, poor sleep quality is accompanied by lower muscle strength and lean body mass and more pronounced anxiety and depressive symptoms (3). These findings support the need for a simple community-based tool that integrates psychological status and physical function to identify current poor sleep quality in middle-aged and older adults with abdominal obesity.

From a public health perspective, abdominal obesity, psychological symptoms, reduced physical function, and poor sleep may coexist in community aging populations but are often assessed separately. A risk-identification approach integrating information already available from psychological and functional assessments could help prioritize individuals for further sleep evaluation. This gap provides the rationale for examining a multidimensional community-oriented model in the present study.

Previous studies have developed prediction models for sleep quality or sleep disorders in different middle-aged and older populations (12, 13). However, the selected predictors vary substantially, and most studies have focused on general older populations or patients with specific chronic diseases. Du et al. developed a sleep quality model in older adults using demographic, health, psychosocial, depressive-symptom, and cognitive variables (12). Zhang et al. developed a sleep disturbance model for older adults with hypertension using variables including depressive symptoms, education, asthma, overweight status, arthritis, and work limitations (13). Models have also been developed in patients with inflammatory bowel disease and systemic lupus erythematosus (14, 15). These models rely mainly on demographic, disease-related, and psychosocial information, with less emphasis on physical function. However, lower muscle strength (16), grip strength and physical performance (17, 18), and multiple dimensions of functional fitness (19) have been associated with sleep quality in older adults. We therefore developed and internally validated a community-oriented nomogram integrating psychological and physical fitness indicators to identify current poor sleep quality among middle-aged and older adults with abdominal obesity, and compared its performance with that of a routine-information model to evaluate the potential added value of multidimensional assessment in aging-related public health practice.

## 2 Methods

### 2.1 Study design and participants

This single-center retrospective cross-sectional study analyzed existing data collected in 2026 at the Huayuan Road Community Health Service Center, Haidian District, Beijing, China. The source dataset comprised 308 community-dwelling middle-aged and older adults who underwent health and physical fitness assessments at the center. Inclusion criteria were: age ≥ 45 years; meeting the criteria for abdominal obesity, i.e., waist circumference ≥ 90 cm for men and ≥ 85 cm for women (20); and the ability to complete the questionnaire assessments and primary physical fitness tests required for the study. Exclusion criteria were documented obstructive sleep apnea, current use of hypnotic medication, and sleep disturbance attributed to a diagnosed medical condition. Other comorbidities were not exclusionary unless they were considered responsible for the sleep disturbance. Habitual snoring and shift-work status were not used as exclusion criteria; participants with these characteristics may therefore have been included. Data screening separately assessed the completeness of Pittsburgh Sleep Quality Index (PSQI) data, duplicate records, and unverifiable key information. The participant selection process is summarized in Figure 1.

**Figure 1:**
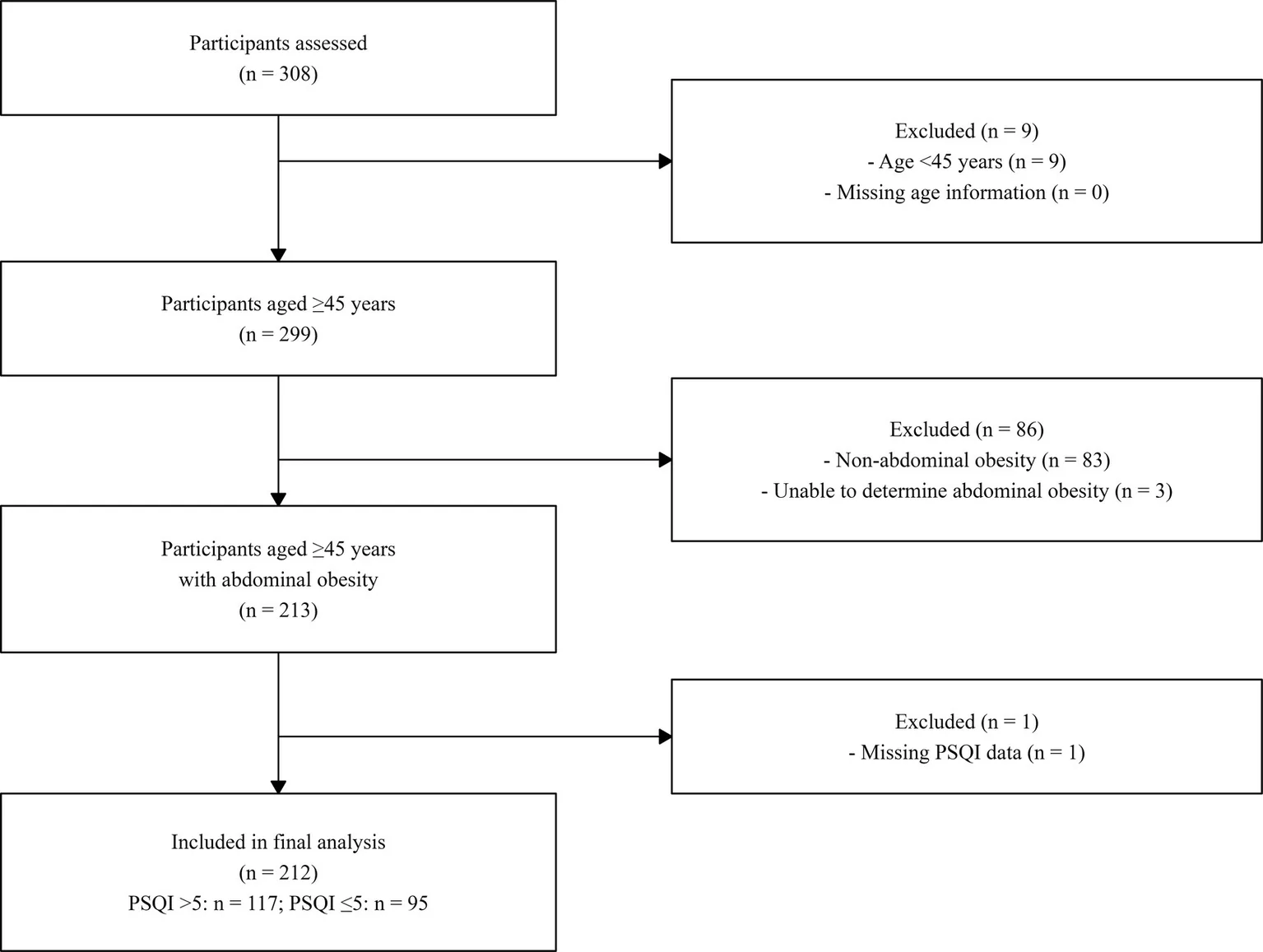
Flowchart of participant selection.

### 2.2 Ethics statement

This study was approved by the Sports Science Experiment Ethics Committee of Beijing Sport University (Approval No. 2026354H) and was conducted in accordance with the Declaration of Helsinki. All participants provided written informed consent at the time of the original assessment. Direct identifiers were removed, and study data were managed using unique participant identifiers.

### 2.3 Assessment of poor sleep quality

This study used the Pittsburgh Sleep Quality Index (PSQI) to assess sleep quality over the previous month (21). The validated Chinese-language version was administered (22). The PSQI comprises seven components: subjective sleep quality, sleep latency, sleep duration, habitual sleep efficiency, sleep disturbances, use of sleeping medication, and daytime dysfunction. Each component is scored from 0 to 3, yielding a total score of 0–21; higher scores indicate poorer sleep quality. According to the threshold proposed by Buysse et al. (21), a PSQI score > 5 was classified as poor sleep quality and a score ≤ 5 as better sleep quality. The model outcome was current poor sleep quality (PSQI > 5) at the time of the survey.

### 2.4 General characteristics

Participant characteristics were collected by trained investigators using a standardized study-specific structured questionnaire and included age, sex, education, marital status, smoking, alcohol consumption, exercise status, physical activity level, number of disease categories, and medication use. Height and weight were measured by trained assessors using a seca 287 ultrasonic measuring station (seca GmbH & Co. KG, Germany). Height was recorded to the nearest 0.1 cm, and weight to the nearest 0.05 kg for values ≤150 kg and 0.1 kg for values >150 kg. Waist circumference was measured using a non-elastic tape at the midpoint between the lower margin of the lowest rib and the upper border of the iliac crest along the midaxillary line. Two measurements were obtained and averaged; measurements were repeated when the prespecified allowable difference was exceeded. Body mass index (BMI) was calculated as weight (kg)/height (m)^2^. Abdominal obesity was defined using the commonly applied waist-circumference thresholds for Chinese adults (20). Waist circumference defined the study population but was not considered as a candidate predictor in the main model.

### 2.5 Measurement of physical and mental function

#### 2.5.1 Depressive symptoms

Depressive symptoms were assessed using the 9-item Patient Health Questionnaire (PHQ-9) (23), which assesses the frequency of depressive symptoms during the previous 2 weeks. The validated Chinese-language version was administered (24). Each item is scored from 0 to 3, yielding a total score of 0–27; higher scores indicate more severe depressive symptoms. The PHQ-9 total score was analyzed as a continuous variable.

#### 2.5.2 Anxiety symptoms

Anxiety symptoms were assessed using the 7-item Generalized Anxiety Disorder scale (GAD-7) (25), which assesses symptom frequency during the previous 2 weeks. The Chinese-language version has demonstrated acceptable psychometric properties in a Chinese clinical population (26). Each item is scored from 0 to 3, yielding a total score of 0–21; higher scores indicate more severe anxiety symptoms. The GAD-7 total score was analyzed as a continuous variable.

#### 2.5.3 Cognitive function

Cognitive function was assessed using the Mini-Mental State Examination (MMSE) (27), which covers orientation, memory, attention and calculation, recall, and language. The culturally adapted Chinese-language version was administered (28). Total scores range from 0 to 30, with higher scores indicating better global cognitive function. The MMSE total score was analyzed as a continuous variable.

#### 2.5.4 Grip strength

Grip strength was used to assess upper-limb muscle strength (29). During testing, participants used a standard grip dynamometer to measure grip strength on their dominant side. Two measurements were taken with an adequate rest period between each, and the higher of the two values was recorded in kilograms (kg). A higher grip strength value indicates better upper-limb muscle strength.

#### 2.5.5 Lower-limb muscle strength

Lower-limb muscle strength was assessed using the 30-s chair stand test (30). Participants sat in the center of a chair and repeatedly performed the movement from a seated position to a fully standing position and back to a seated position without using their arms for support. The number of correct repetitions completed within 30 s was recorded. A higher number of repetitions indicates better lower-limb muscle strength and functional performance.

#### 2.5.6 Flexibility

Lower-body flexibility was assessed using the chair sit-and-reach test (31). During the test, participants sat on the front edge of a chair, extended one leg forward while keeping the heel on the ground, and slowly reached forward with both hands toward the toes; the distance between the fingertips and the toes was recorded. The test was performed twice on each side, and the maximum value among the measurements was used for analysis, with units in cm; a higher value indicates better flexibility.

#### 2.5.7 Cardiorespiratory endurance

Cardiorespiratory endurance was assessed using the 2-min step test (32, 33). During the test, participants performed continuous stepping in place for 2 min while raising their knees to a specified height; the number of correctly completed steps within the specified time was recorded. A higher number of completed steps indicates better cardiorespiratory endurance.

#### 2.5.8 Functional mobility

Functional mobility was assessed using the 8-Foot Up-and-Go test (8UG), a modified version of the conventional TUG (34). Participants stood up from a chair, walked as quickly and safely as possible around a cone placed 2.44 m from the chair, turned around, walked back, and sat down again; the time required to complete the entire process was recorded. The test was conducted twice, and the shorter time was used for analysis, with results reported in seconds; a shorter completion time indicates better functional mobility.

### 2.6 Statistical analysis

Statistical analyses were performed using R version 4.6.1. Categorical variables were summarized as frequencies and percentages, and continuous variables as means with standard deviations or medians with interquartile ranges, as appropriate. Between-group comparisons used Welch’s independent-samples t-test or the Mann–Whitney U test for continuous variables and Pearson’s *χ*^2^ test or Fisher’s exact test for categorical variables.

Because this retrospective study used an existing dataset, all eligible participants were included; consequently, recruitment was not determined by a formal *a priori* sample-size calculation. Conventional *post hoc* power analysis was not performed because the study objective was prediction rather than null-hypothesis testing. Instead, a *post hoc* sample-size adequacy assessment was conducted using the Riley framework for binary-outcome prediction models (35). Based on 10 candidate predictor parameters, an outcome prevalence of 0.552, and the observed Nagelkerke R^2^ of 0.309, the estimated minimum sample was 381 participants, including approximately 211 events. The available sample of 212 participants with 117 events did not meet all conservative criteria for definitive prediction-model development; the model was therefore interpreted as exploratory, and bootstrap resampling was used to quantify optimism rather than to compensate for sample-size limitations.

The definitions and coding of the 10 candidate predictors are summarized in Supplementary Table S1. The 10 candidate predictors were prespecified before model development based on prior evidence, clinical relevance, community feasibility, measurement burden, and avoidance of conceptual redundancy; Table 1 group-comparison *p* values were not used for candidate selection. The pool comprised age and sex; depressive symptoms, anxiety symptoms, and cognitive function; and one representative test for each functional domain of upper- and lower-limb strength, flexibility, cardiorespiratory endurance, and mobility. Other general variables were used for descriptive analyses or the routine-information comparator. BMI and waist measures were excluded from the primary candidate pool because abdominal obesity defined eligibility, while additional fitness measures were omitted because their constructs were already represented by the selected domain-specific tests. This restriction was intended to preserve interpretability and control model complexity. LASSO logistic regression was used to select candidate predictors (36). Continuous variables were standardized before modeling. The penalty parameter was selected using 100 repetitions of stratified 10-fold cross-validation. The primary rule was λ_min_, which minimizes the cross-validated binomial deviance; the predictor set at λ_₁SE_ was also examined. Variable-selection stability was evaluated by repeating LASSO in 1,000 bootstrap resamples. Predictors with nonzero coefficients at λ_₁SE_ were entered simultaneously into an unpenalized multivariable logistic regression model, from which the nomogram was constructed.

**Table 1:** Baseline characteristics according to sleep quality status.

Characteristic	Total (*n* = 212)	PSQI ≤ 5 (*n* = 95)	PSQI > 5 (*n* = 117)	*p*
Demographic and lifestyle characteristics				
Age, years	65.81 ± 6.86	64.59 ± 7.41	66.79 ± 6.25	0.022
Sex				0.517
Male	60 (28.3)	29 (30.5)	31 (26.5)	
Female	152 (71.7)	66 (69.5)	86 (73.5)	
Education level				0.565
Elementary school or below	6 (2.8)	2 (2.1)	4 (3.4)	
Junior high school	41 (19.3)	18 (18.9)	23 (19.7)	
High school/college	105 (49.5)	45 (47.4)	60 (51.3)	
Bachelor’s degree	50 (23.6)	23 (24.2)	27 (23.1)	
Postgraduate	10 (4.7)	7 (7.4)	3 (2.6)	
Marital status				0.112
Unmarried	5 (2.4)	0 (0.0)	5 (4.3)	
Married	197 (92.9)	91 (95.8)	106 (90.6)	
Divorced/widowed	10 (4.7)	4 (4.2)	6 (5.1)	
Smoking status				0.221
Never smoked	185 (87.3)	80 (84.2)	105 (89.7)	
Former smoker	21 (9.9)	13 (13.7)	8 (6.8)	
Current smoker	6 (2.8)	2 (2.1)	4 (3.4)	
Alcohol consumption				0.193
Never drinker	141 (66.5)	68 (71.6)	73 (62.4)	
Former drinker	3 (1.4)	0 (0.0)	3 (2.6)	
Current drinker	68 (32.1)	27 (28.4)	41 (35.0)	
Exercise status				0.106
No exercise	60 (28.3)	20 (21.1)	40 (34.2)	
Occasional exercise	74 (34.9)	36 (37.9)	38 (32.5)	
Regular exercise	78 (36.8)	39 (41.1)	39 (33.3)	
Physical activity level				0.523
High	43 (20.3)	19 (20.0)	24 (20.5)	
Moderate	117 (55.2)	56 (58.9)	61 (52.1)	
Low	52 (24.5)	20 (21.1)	32 (27.4)	
Number of disease categories				0.233
0 diseases	10 (4.7)	7 (7.4)	3 (2.6)	
1 disease	28 (13.2)	11 (11.6)	17 (14.5)	
≥2 diseases	174 (82.1)	77 (81.1)	97 (82.9)	
Number of medication categories				0.765
0 medications	51 (24.1)	24 (25.3)	27 (23.1)	
1–4 medications	135 (63.7)	61 (64.2)	74 (63.2)	
≥5 medications	26 (12.3)	10 (10.5)	16 (13.7)	
Psychological and cognitive characteristics				
PHQ-9 score	3.00 (1.00, 5.00)	2.00 (1.00, 3.00)	4.00 (2.00, 6.00)	<0.001
GAD-7 score	3.00 (1.00, 5.00)	2.00 (1.00, 3.00)	4.00 (2.00, 6.00)	<0.001
MMSE score	27.00 (25.00, 28.00)	27.00 (25.00, 28.00)	27.00 (25.00, 28.00)	0.387
Anthropometric and physiological characteristics				
Body mass index, kg/m^2^	25.65 ± 2.98	25.68 ± 2.94	25.62 ± 3.02	0.871
Waist circumference, cm	91.95 (87.00, 96.53)	92.00 (87.00, 97.75)	91.50 (87.00, 96.50)	0.959
Mean arm circumference, cm	27.94 ± 2.42	28.20 ± 2.45	27.73 ± 2.38	0.168
Body fat percentage, %	33.07 ± 6.93	32.66 ± 7.78	33.39 ± 6.18	0.456
Systolic blood pressure, mmHg	133.21 ± 18.56	133.87 ± 16.45	132.67 ± 20.17	0.633
Diastolic blood pressure, mmHg	79.24 ± 10.29	78.58 ± 9.57	79.78 ± 10.85	0.394
Functional fitness				
Grip strength, kg	28.76 ± 7.99	30.00 ± 8.70	27.74 ± 7.25	0.045
30-s chair stand, repetitions	16.14 ± 4.40	16.99 ± 4.21	15.44 ± 4.45	0.010
30-s arm curl, repetitions	26.00 (22.75, 29.00)	27.00 (23.00, 30.50)	24.00 (22.00, 27.00)	0.002
Back scratch, cm	−5.78 ± 10.35	−5.76 ± 9.34	−5.80 ± 11.15	0.975
Chair sit-and-reach, cm	5.84 ± 12.17	7.90 ± 11.81	4.17 ± 12.25	0.026
One-leg stance with eyes closed, s	3.21 (2.27, 4.81)	3.44 (2.63, 4.83)	3.13 (2.01, 4.48)	0.033
2-min step test, steps	100.61 ± 17.17	103.77 ± 14.27	98.04 ± 18.88	0.013
8UG, s	6.87 (6.16, 7.63)	6.83 (6.06, 7.49)	6.98 (6.19, 7.77)	0.174

Data are presented as mean ± standard deviation, median (Q1, Q3), or *n* (%), as appropriate. *p* values were calculated using Welch’s independent-samples *t*-test or the Mann–Whitney U test for continuous variables and Pearson’s *χ*^2^ test or Fisher’s exact test for categorical variables, as appropriate. PSQI, Pittsburgh Sleep Quality Index; PHQ-9, Patient Health Questionnaire-9; GAD-7, Generalized Anxiety Disorder-7; MMSE, Mini-Mental State Examination; BMI, body mass index.

Discrimination was evaluated using the area under the receiver operating characteristic curve (AUC). The Youden index determined the apparent optimal cutoff and corresponding sensitivity and specificity (37). Calibration was evaluated using a calibration plot, calibration intercept, and calibration slope (38, 39), and overall prediction error was quantified using the Brier score. After the seven-predictor structure was fixed, a separate 1,000-resample bootstrap procedure estimated optimism-corrected model performance (40). Decision curve analysis (DCA) evaluated theoretical net benefit across threshold probabilities.

A routine-information model including age, sex, number of disease categories, current alcohol consumption, and exercise level was compared with the final model in the same participants. The comparator was constructed as a parsimonious, low-burden benchmark that could be applied without psychological scales or fitness testing. Its structure was fixed before the reported head-to-head comparison and was not modified thereafter. Age and sex represented core demographic information, number of disease categories represented comorbidity burden, and current alcohol consumption and exercise level represented distinct lifestyle domains. Education was outside the intended core health-information set; BMI was not included because the cohort was restricted to participants with abdominal obesity; smoking and physical activity were not added to avoid expanding overlapping lifestyle constructs; and medication use was not included alongside disease burden to limit related information and model complexity. The comparison therefore estimates added performance relative to this specific pragmatic benchmark rather than to every possible routine-information model. Apparent AUCs were compared using a paired DeLong test (41), and 95% confidence intervals for other performance differences were estimated using 1,000 paired bootstrap resamples. LASSO was implemented with glmnet, the nomogram and calibration analyses with rms, ROC analysis with pROC, and DCA with dcurves. All tests were two-sided, and *p* < 0.05 was considered statistically significant. Further modeling details are provided in Supplementary methods S1.

## 3 Results

### 3.1 Study population selection

The source dataset initially included 308 middle-aged and older adults assessed at the Huayuan Road Community Health Service Center. Of these, 9 were excluded because they were younger than 45 years old, 86 were excluded because they did not meet the criteria for abdominal obesity or could not be classified as such, and 1 was excluded due to missing PSQI data. Ultimately, 212 participants were included in the analysis. The participant selection process is shown in Figure 1. The mean age of the included participants was 65.81 ± 6.86 years; 152 were female, accounting for 71.7%. Among them, 117 participants were classified as having poor sleep quality (PSQI > 5), accounting for 55.2%. A comparison of the baseline characteristics of participants with different sleep quality is shown in Table 1.

Compared with participants with better sleep quality, those with poor sleep quality were older (66.79 ± 6.25 vs. 64.59 ± 7.41 years) and had higher PHQ-9 and GAD-7 scores. They also had lower grip strength, fewer 30-s chair stand and 30-s arm curl repetitions, shorter chair sit-and-reach distance and eyes-closed single-leg stance time, and a lower 2-min step count (all *p* < 0.05).

### 3.2 Selection of predictors

The 10 candidate predictors were age, sex, PHQ-9 score, GAD-7 score, MMSE total score, grip strength, 30-s chair stand repetitions, chair sit-and-reach distance, 2-min step count, and 8UG time. LASSO selected seven predictors. At λ_min_, age, PHQ-9 score, GAD-7 score, grip strength, 30-s chair stand repetitions, chair sit-and-reach distance, and 2-min step count had nonzero coefficients; the same predictors were retained at λ_₁SE_ (Figures 2, 3). Selection frequencies and coefficient-direction consistency across 1,000 bootstrap resamples are reported in Table 2, with additional details provided in Supplementary Figure S1 and Supplementary Table S2.

**Figure 2:**
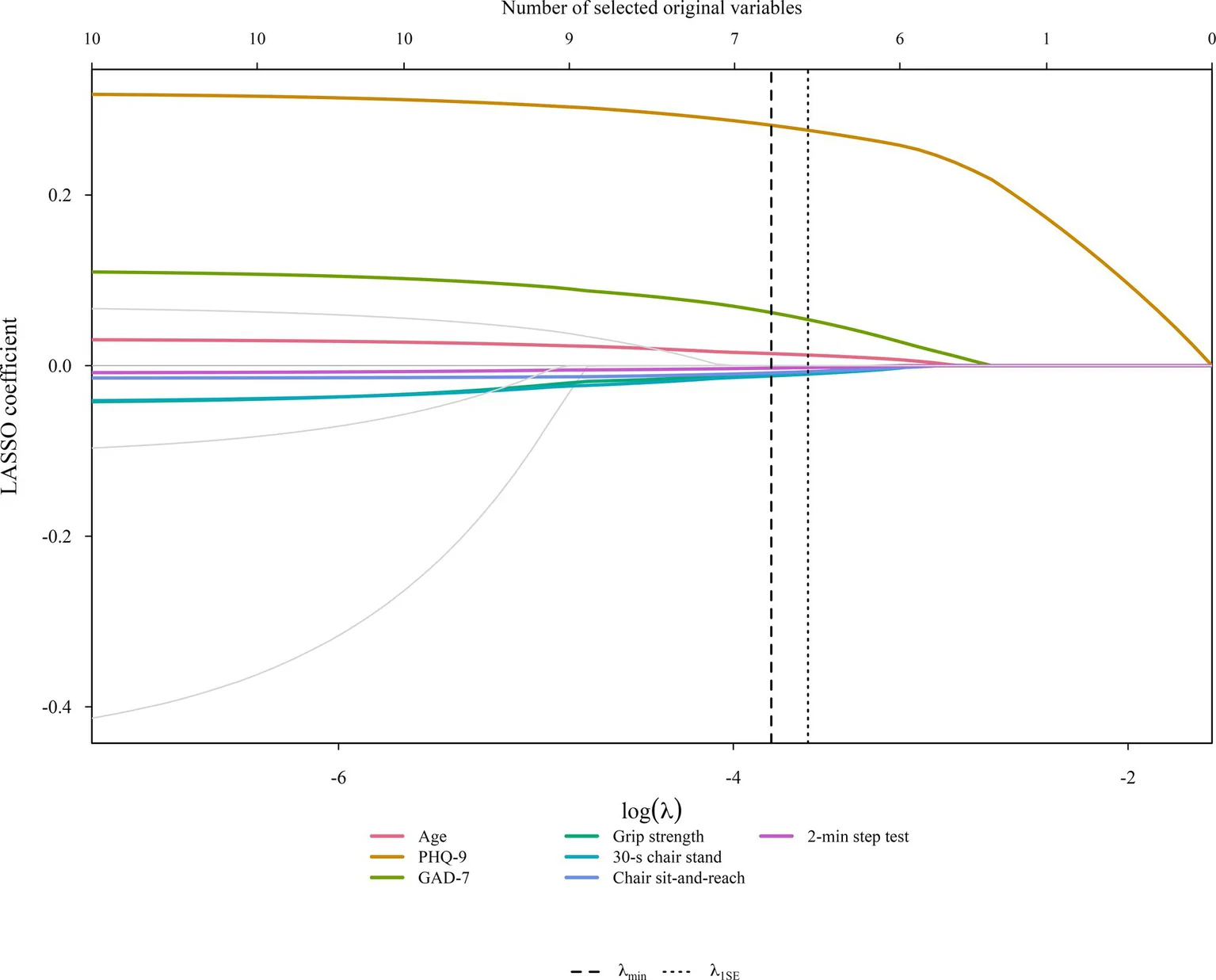
LASSO coefficient profiles of the 10 candidate predictors. Each curve represents the change in the standardized coefficient of a candidate predictor as the penalty parameter (λ) increases. Vertical dashed and dotted lines indicate λ_min_ and λ_₁SE-_, respectively. LASSO, least absolute shrinkage and selection operator; λ_min_, the value of λ that minimizes the cross-validated binomial deviance; λ_₁SE-_, the largest value of λ within one standard error of the minimum.

**Figure 3:**
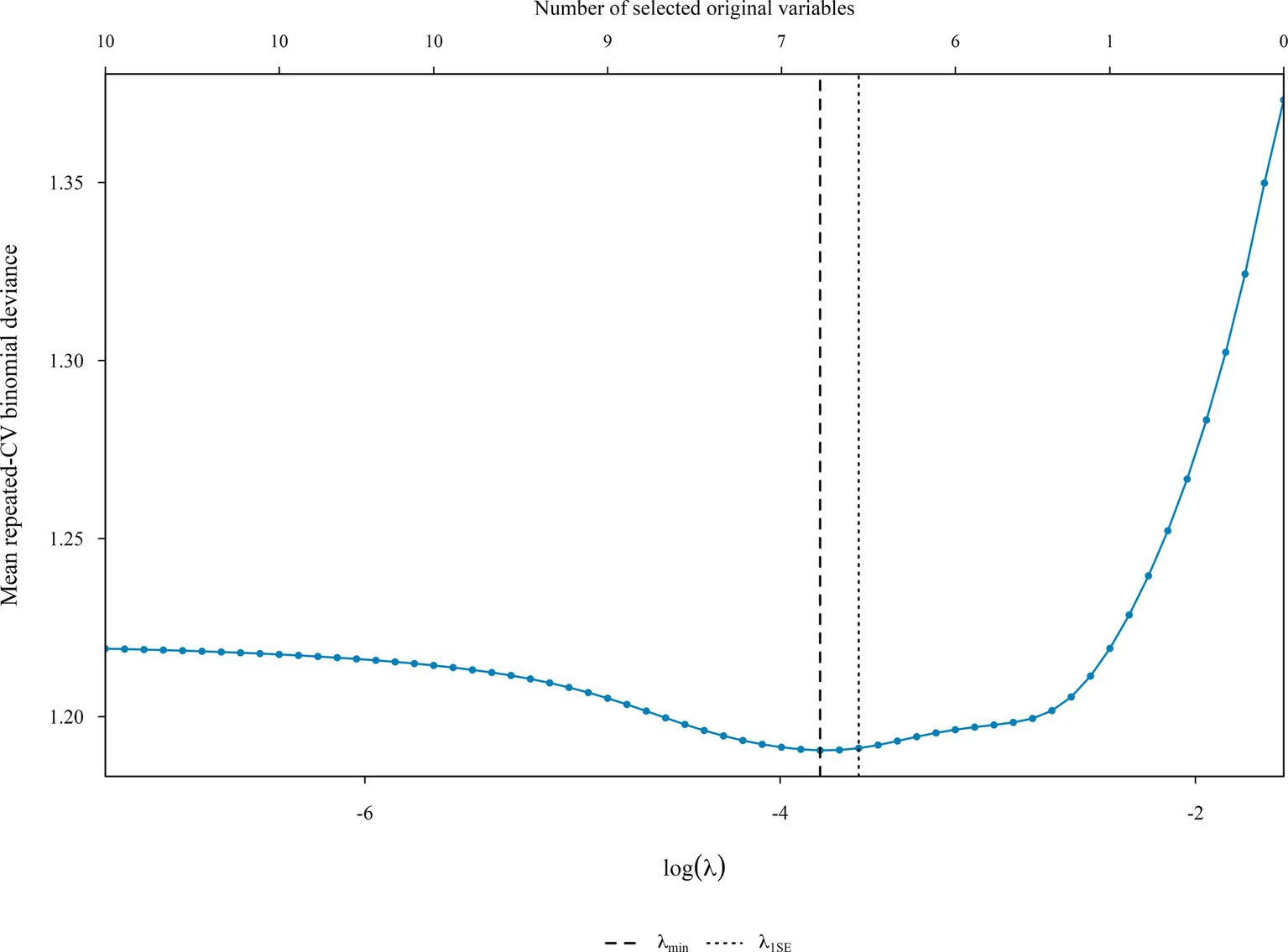
Selection of the optimal penalty parameter using repeated stratified 10-fold cross-validation. The optimal penalty parameter was determined using the minimum-error criterion (λ_min_), with λ_₁SE_ additionally examined as a more parsimonious criterion. Seven predictors were retained under both criteria. λ_min_, the value of λ that minimizes the cross-validated binomial deviance; λ_₁SE_, the largest value of λ within one standard error of the minimum.

**Table 2:** Variable selection using LASSO and bootstrap stability analysis.

Variables	*β at λ_min_*	*β at λ_₁SE_*	bootstrap selection, %	Direction consistency, %
Demographic characteristics				
Age	0.014	0.012	72.8	96.6
Sex	0	0	34.6	—
Mental and physical functioning variables				
PHQ-9	0.282	0.276	100.0	100.0
GAD-7	0.062	0.054	83.8	99.9
MMSE	0	0	50.3	83.9
Grip strength	−0.010	−0.008	68.3	99.0
30-s chair stand	−0.012	−0.010	62.3	96.0
Chair sit-and-reach	−0.009	−0.007	73.2	98.4
2-min step test	−0.003	−0.002	59.9	92.5
8UG	0	0	36.7	59.9

At λ_min_ (λ = 0.0222), seven variables had non-zero coefficients: age, PHQ-9, GAD-7, grip strength, 30-s chair stand, chair sit-and-reach, and 2-min step test. The same seven variables were retained at λ_₁SE_ (λ = 0.0267). The bootstrap selection frequency was calculated from 1,000 resamples. Direction consistency refers to the proportion of selected bootstrap samples in which the coefficient had the same direction as in the full-sample model; it is not reported for sex because the categorical term was not retained in the full-sample model. GAD-7, Generalized Anxiety Disorder-7; MMSE, Mini-Mental State Examination; PHQ-9, Patient Health Questionnaire-9.

The seven LASSO-selected predictors were entered simultaneously into the multivariable logistic regression model. Each 1-point increase in PHQ-9 score was associated with a 37.4% increase in the adjusted odds of current poor sleep quality (OR = 1.374, 95% CI, 1.164–1.621; *p* < 0.001). Associations for age, GAD-7 score, grip strength, 30-s chair stand repetitions, chair sit-and-reach distance, and 2-min step count did not reach statistical significance (Table 3). No substantial multicollinearity was detected; supporting correlation and model-diagnostic results are provided in Supplementary Figure S2 and Supplementary Table S3.

**Table 3:** Multivariable logistic regression analysis of factors associated with poor sleep quality.

Variables	*β*	SE	Wald *χ*^2^	OR	95% CI	*p*	VIF
Demographic characteristic							
Age, per 5-year increase	0.115	0.123	0.874	1.122	0.882–1.428	0.348	1.103
Mental and functioning variables							
PHQ-9, per 1-point increase	0.318	0.085	13.996	1.374	1.164–1.621	<0.001	1.265
GAD-7, per 1-point increase	0.110	0.072	2.334	1.116	0.969–1.286	0.127	1.242
Grip strength, per 5-kg increase	−0.114	0.104	1.202	0.892	0.728–1.093	0.270	1.147
30-s chair stand, per 1-repetition increase	−0.023	0.040	0.331	0.977	0.903–1.057	0.564	1.298
Chair sit-and-reach, per 5-cm increase	−0.082	0.070	1.372	0.921	0.803–1.057	0.241	1.143
2-min step test, per 10-repetition increase	−0.063	0.107	0.347	0.939	0.761–1.159	0.559	1.295

Poor sleep quality was defined as a PSQI score greater than 5 (coded as 1), with a PSQI score of 5 or less coded as 0. All seven variables selected by LASSO were included simultaneously. CI, confidence interval; GAD-7, Generalized Anxiety Disorder-7; OR, odds ratio; PHQ-9, Patient Health Questionnaire-9; PSQI, Pittsburgh Sleep Quality Index; VIF, variance inflation factor.

### 3.3 Construction of the nomogram model

A nomogram for estimating the probability of current poor sleep quality was constructed from the final multivariable logistic regression model (Figure 4). It included age, PHQ-9 score, GAD-7 score, grip strength, 30-s chair stand repetitions, chair sit-and-reach distance, and 2-min step count. Predictor-specific points are summed to obtain a total score, which corresponds to the estimated probability of current poor sleep quality.

**Figure 4:**
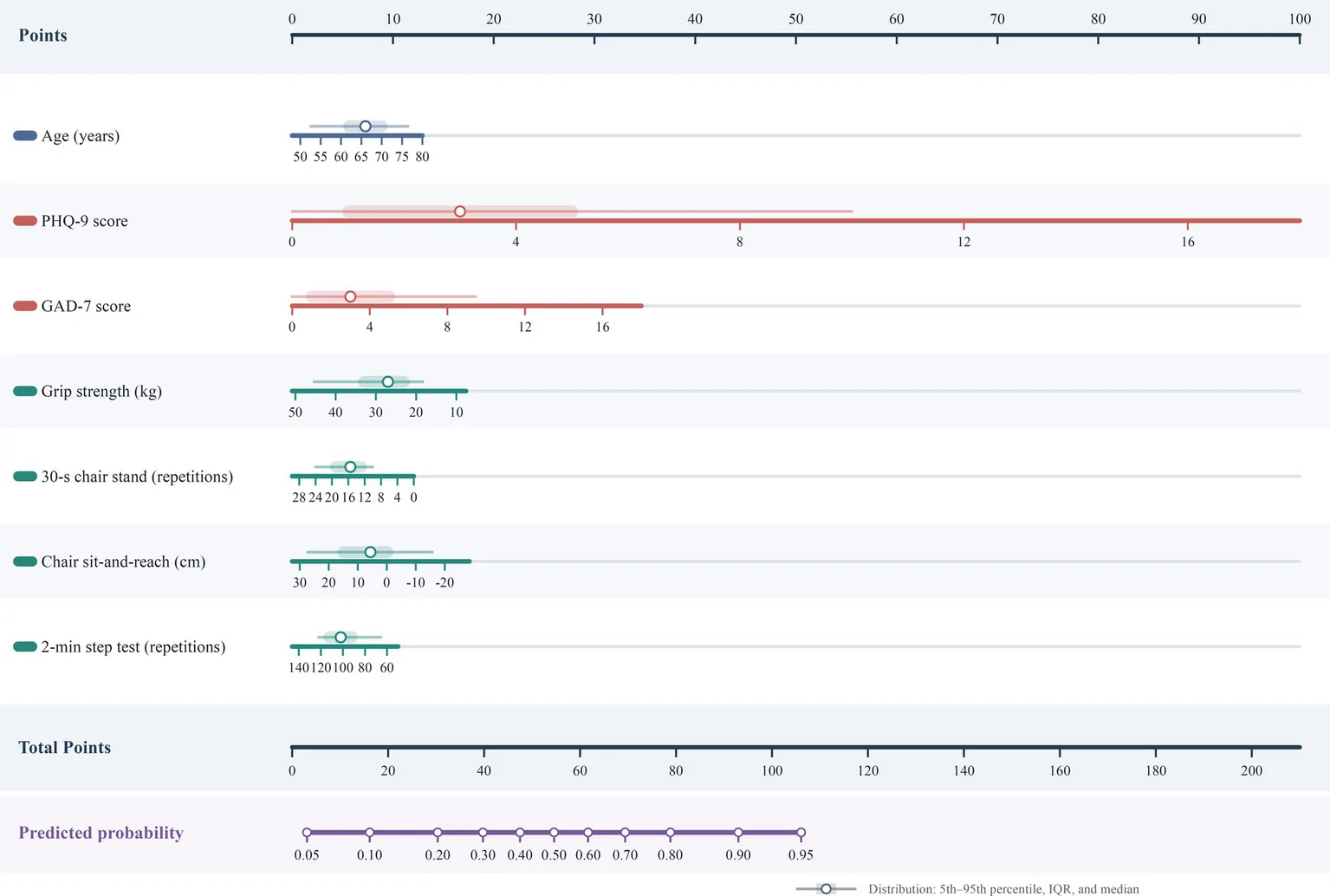
Nomogram for estimating the probability of current poor sleep quality in middle-aged and older adults with abdominal obesity. For each predictor, a point value is assigned by projecting its observed value onto the “Points” scale. The individual point values are summed to obtain the total score, which corresponds to the estimated probability of current poor sleep quality. PHQ-9, Patient Health Questionnaire-9; GAD-7, Generalized Anxiety Disorder-7.

For example, a 65-year-old participant with a PHQ-9 score of 8, a GAD-7 score of 8, a grip strength of 30 kg, 16 30-s chair stands, a chair sit-and-reach distance of 0 cm, and a 2-min step test with 100 steps has scores of approximately 6.8, 50.0, 17.3, 9.0, 5.5, 9.7, and 4.9, respectively, for a total score of approximately 103.2. The estimated probability of poor sleep quality for this participant is approximately 91% (Figure 4).

### 3.4 Evaluation of the nomogram model

The apparent AUC was 0.779 (95% CI, 0.718–0.840), and the optimism-corrected AUC after 1,000 bootstrap resamples was 0.751, indicating moderate discrimination (Figure 5A). The optimism-corrected calibration intercept, calibration slope, and Brier score were 0.002, 0.858, and 0.206, respectively (Figure 5B and Supplementary Table S4). In the development sample, the model provided greater theoretical net benefit than the “Treat all” and “Treat none” strategies across threshold probabilities of 13–80% (Figure 6).

**Figure 5:**
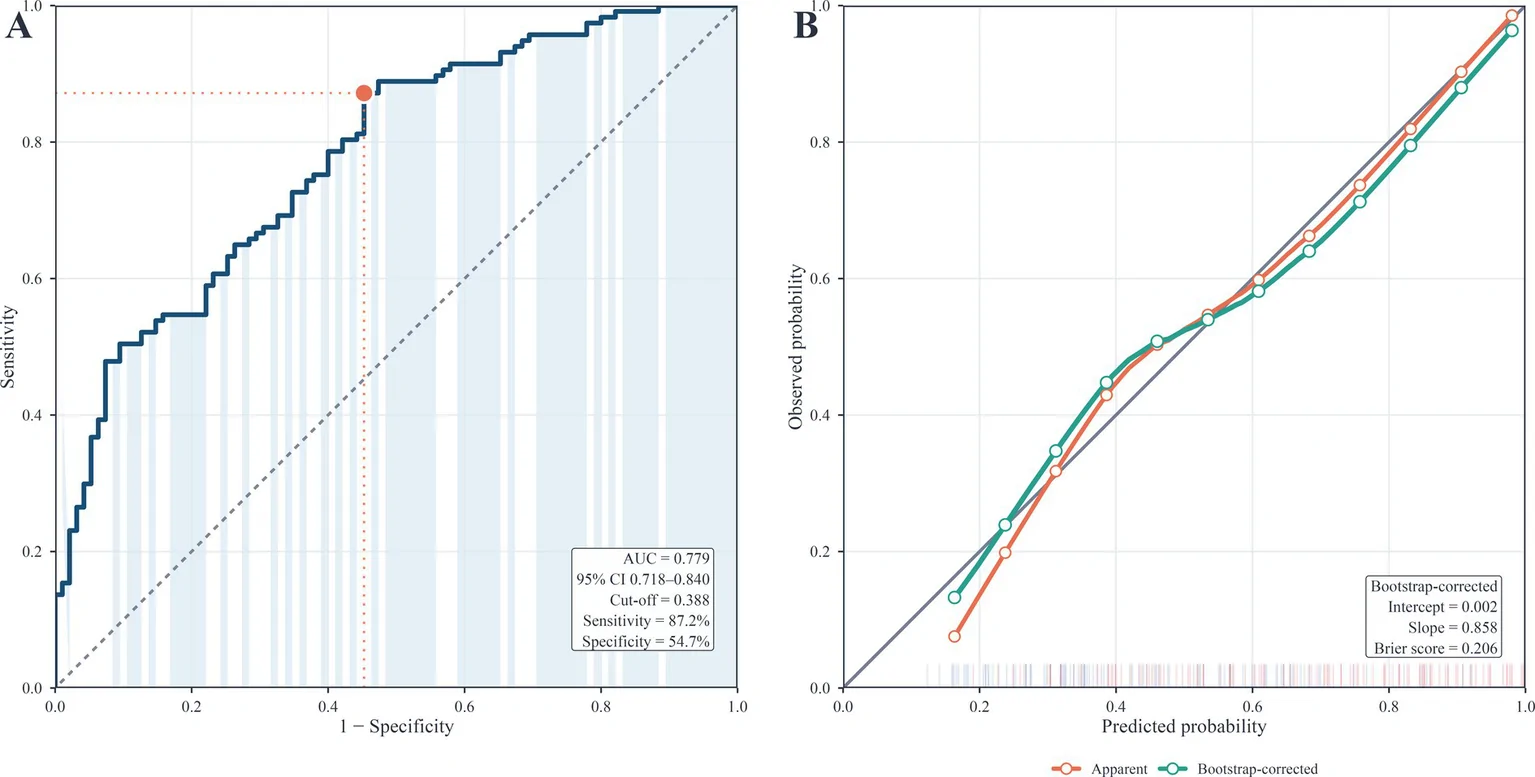
Discrimination and calibration of the final model for identifying current poor sleep quality. **(A)** Receiver operating characteristic curve of the final model. The apparent area under the curve was 0.779 [95% confidence interval (CI), 0.718–0.840]. The highlighted point indicates the optimal probability threshold determined using the Youden index. **(B)** Calibration curves of the final model. The gray diagonal line indicates perfect calibration, the coral curve indicates apparent calibration, and the teal curve indicates optimism-corrected calibration based on 1,000 bootstrap resamples. Short vertical ticks along the x-axis show the distribution of predicted probabilities: blue ticks represent participants with Pittsburgh Sleep Quality Index (PSQI) scores ≤5, and red ticks represent those with PSQI scores >5. Abbreviation: AUC, area under the curve.

**Figure 6:**
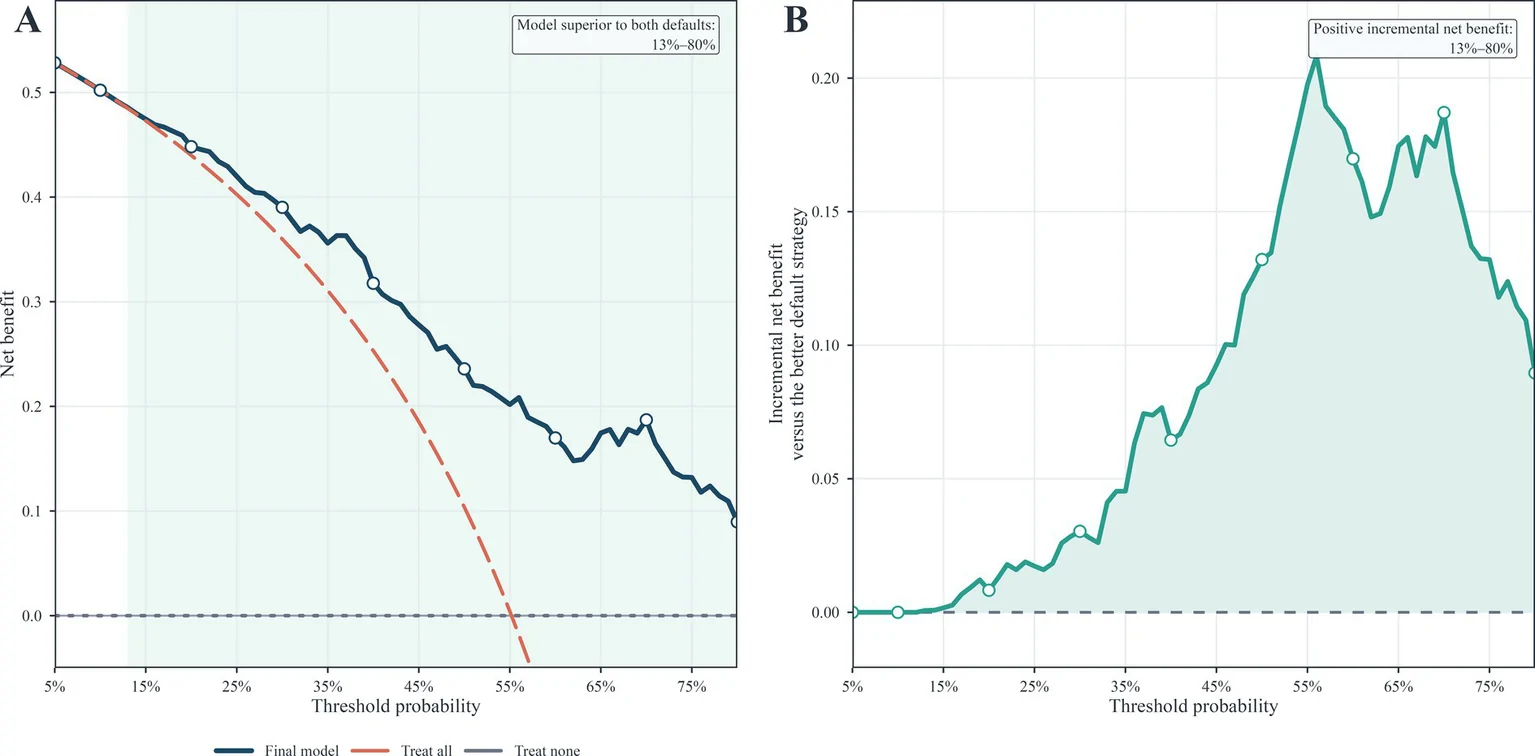
Decision curve analysis of the final prediction model. **(A)** Net benefit of the final model, the “Treat all” strategy, and the “Treat none” strategy across threshold probabilities ranging from 5 to 80%. **(B)** Incremental net benefit of the final model relative to the better default strategy. The shaded region indicates the threshold range in the development sample in which the final model showed greater theoretical net benefit than both default strategies.

### 3.5 Comparison of model performance

Compared with the routine-information model, the final model had higher apparent and optimism-corrected AUCs and a lower Brier score (Table 4). Coding and coefficient estimates for the routine-information model are provided in Supplementary Table S5. The apparent AUCs were 0.648 (95% CI, 0.574–0.722) and 0.779 (95% CI, 0.718–0.840), respectively, with a difference of 0.131 (95% CI, 0.050–0.213; *p* = 0.002). The corresponding optimism-corrected AUCs were 0.608 and 0.751, and the optimism-corrected Brier scores were 0.245 and 0.206. In the development sample, the final model showed greater theoretical net benefit than the routine-information model across threshold probabilities of 13–80% (Figure 7; Supplementary Table S6).

**Table 4:** Comparison of predictive performance between the routine-information model and the final model.

Performance measure	Routine-information model	Final model	Difference (Final − Routine)	95% CI for difference	*p*
Discrimination					
AUC, apparent	0.648 (0.574–0.722)	0.779 (0.718–0.840)	0.131	0.050–0.213	0.002
AUC, optimism-corrected	0.608	0.751	0.143	0.063–0.222	—
Overall prediction error					
Brier score, apparent	0.230	0.190	−0.040	−0.067 to −0.016	—
Brier score, optimism-corrected	0.245	0.206	−0.039	−0.069 to −0.006	—
Scaled Brier score, apparent	0.070	0.230	0.160	0.063–0.274	—
Scaled Brier score, optimism-corrected	0.014	0.172	0.158	0.020–0.280	—
Calibration					
Calibration intercept, optimism-corrected	0.000	0.002	0.002	−0.185–0.185	—
Calibration slope, optimism-corrected	0.723	0.858	0.135	−0.374–0.726	—
Explained variation					
Nagelkerke *R*^2^, apparent	0.098	0.309	0.211	0.084–0.343	—
Nagelkerke *R*^2^, optimism-corrected	−0.010	0.235	0.245	0.033–0.884	—
Model fit					
AIC	289.5	251.9	−37.6	—	—
BIC	313.0	278.8	−34.3	—	—

Differences were calculated as the final model minus the routine-information model. The apparent AUC difference was tested using the paired DeLong method; confidence intervals for other performance differences were estimated from 1,000 paired bootstrap resamples. AIC, Akaike information criterion; AUC, area under the receiver operating characteristic curve; BIC, Bayesian information criterion; CI, confidence interval.

**Figure 7:**
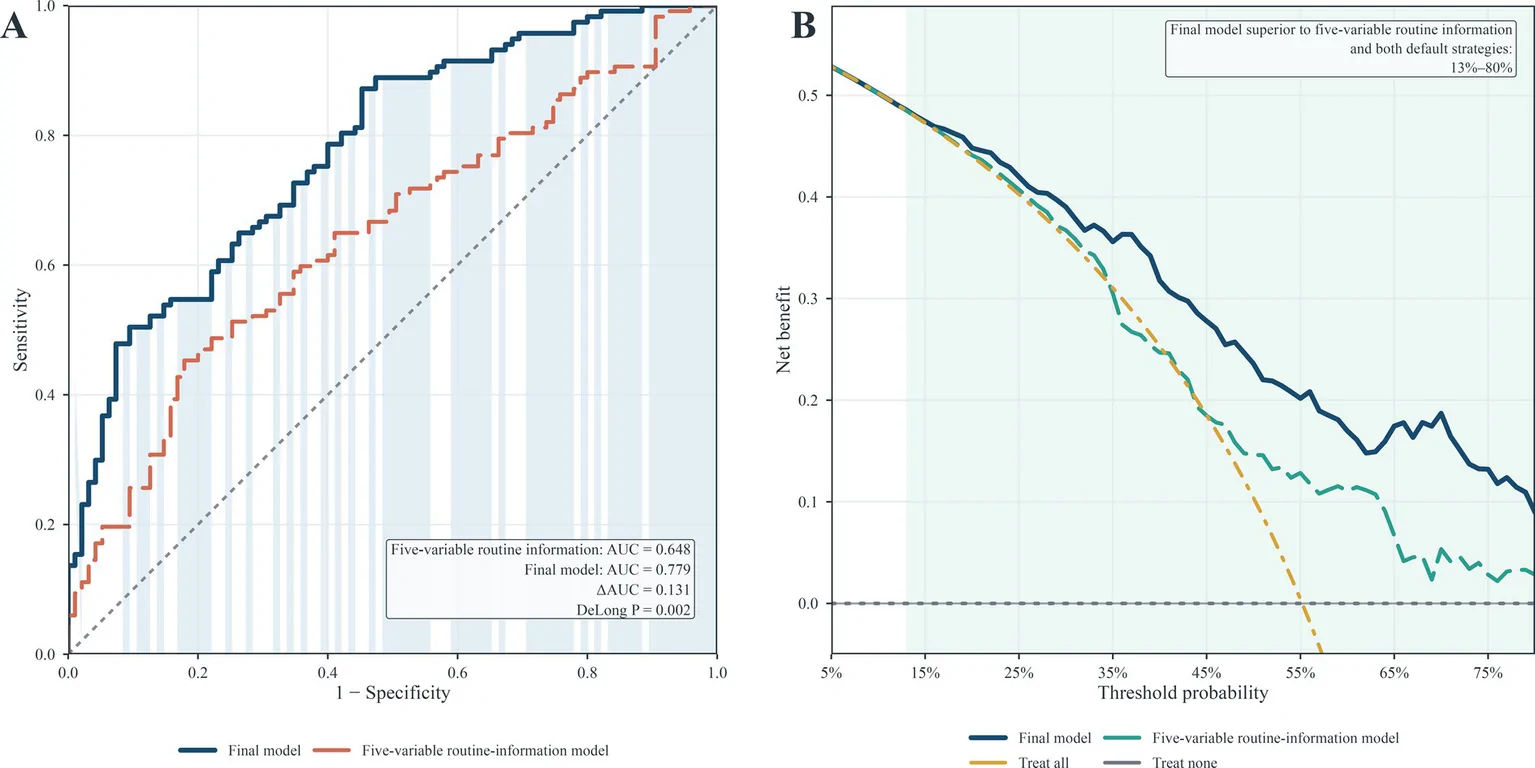
Comparison of predictive performance between the routine-information model and the final model. **(A)** Receiver operating characteristic (ROC) curves of the two models. **(B)** Decision curve analysis comparing the theoretical net benefits in the development sample of the two models with the “Treat all” and “Treat none” strategies. The routine-information model included age, sex, number of disease categories, alcohol consumption, and exercise status. AUC, area under the curve; CI, confidence interval.

## 4 Discussion

This study developed a nomogram for identifying current poor sleep quality using psychological and physical fitness information from 212 community-dwelling middle-aged and older adults with abdominal obesity; 55.2% had a PSQI score > 5. LASSO retained age, PHQ-9 score, GAD-7 score, grip strength, 30-s chair stand repetitions, chair sit-and-reach distance, and 2-min step count. PHQ-9 score was associated with current poor sleep quality in the multivariable model. The apparent AUC was 0.779, and the optimism-corrected AUC was 0.751. Overall performance exceeded that of the routine-information model.

In this study, 55.2% of participants had poor sleep quality, exceeding the pooled prevalence reported among community-dwelling older adults. Canever et al. (1) estimated a prevalence of approximately 40% in a meta-analysis of nearly 1 million older adults from 36 countries, while Fu et al. (2) also reported that sleep disturbances were common but heterogeneous across studies. The higher detection rate in our sample may partly reflect its composition. All participants had abdominal obesity, and 71.7% were women. Chinese population-based research has associated poor sleep quality with abdominal obesity, with possible sex-specific differences (8), while systematic reviews have linked shorter sleep duration with central obesity in adults and obesity in older adults (6, 7). The observed 55.2% should therefore be interpreted as the burden within this specific sample rather than as a prevalence estimate for the general community. Because existing sleep-quality models mainly concern general older populations or specific patient groups, including people with hypertension (12, 13), the present population remains relevant for targeted risk identification.

Abdominal obesity and sleep disturbance may be linked through interacting metabolic, inflammatory, behavioral, and neuroendocrine pathways. Visceral adiposity is associated with inflammation and oxidative stress (9), whereas experimental sleep restriction can alter leptin-related regulation and impair adipose-tissue insulin signaling (10, 11). These pathways provide a possible biological context for the clustering of poor sleep, psychological symptoms, and impaired physical function in adults with abdominal obesity. However, they were not directly evaluated in this study and should not be interpreted as demonstrated causal mechanisms.

Psychological symptoms provided substantial information in the model. Participants with a PSQI score > 5 had higher PHQ-9 and GAD-7 scores, and both were retained by LASSO. In the multivariable model, each 1-point increase in PHQ-9 score was associated with higher odds of current poor sleep quality (OR = 1.374), whereas the adjusted association with GAD-7 score was not statistically significant. Previous studies have likewise associated depressive symptoms (42), anxiety symptoms (4), and obesity accompanied by anxiety and depression (3) with poorer sleep quality. Emotional arousal, altered stress regulation, and inflammatory responses may contribute to the observed overlap, while impaired sleep may also coexist with worsening negative affect (43, 44). The contribution of PHQ-9 should therefore be interpreted as a marker of current poor sleep quality, not evidence that depressive symptoms are an independent cause. Moreover, PHQ-9 Item 3 assesses sleep disturbance and overlaps conceptually with the PSQI (21, 23). Because item-level PHQ-9 data were unavailable, we could not refit the model after excluding this item. This overlap may have inflated the estimated contribution of PHQ-9, overall model performance, and the apparent improvement over the routine-information model; these findings should therefore be interpreted cautiously.

Physical function constituted another dimension of the predictor set. Participants with poor sleep quality performed worse in grip strength, 30-s chair stand, chair sit-and-reach, and 2-min step tests, and all four measures were retained by LASSO. This pattern is consistent with evidence linking poorer sleep with lower muscle strength (16), poorer physical performance (17), grip strength (18), and multiple dimensions of functional fitness (19). These findings may reflect reciprocal links between sleep quality and physical function, including fatigue and lower physical activity, although these pathways were not examined in the present study (16–19). Exercise interventions have also improved subjective sleep quality in middle-aged and older adults (45–47). Nevertheless, the 95% CIs for all four physical fitness measures crossed 1 in the multivariable model. The findings therefore do not establish any single measure as an independent risk factor; rather, the measures collectively capture complementary aspects of physical function relevant to identifying current poor sleep quality.

Not all seven LASSO-selected predictors reached conventional statistical significance in the multivariable logistic regression model. Except for PHQ-9, the 95% CIs for age, GAD-7, and the four physical fitness measures crossed 1. This does not imply that these predictors contain no model information. LASSO retains variables according to their contribution to penalized model fit and prediction error rather than an individual *p* < 0.05 criterion (36); variable selection and coefficient testing therefore address different questions. Across 1,000 bootstrap resamples, PHQ-9, GAD-7, age, and chair sit-and-reach were selected in more than 70% of samples, and the other retained physical fitness measures in more than 50%, with generally consistent coefficient directions. Age may provide limited independent information after accounting for mood, muscle strength, and functional capacity, despite its association with sleep problems in previous research (2). The seven variables should therefore be interpreted as a combined predictor set rather than as seven independent risk factors. For prediction models, overall discrimination, calibration, and prediction error are more informative than individual *p* values (48), consistent with the present focus on risk identification rather than causal inference.

Integrating psychological and physical fitness information improved identification of current poor sleep quality compared with the parsimonious routine-information benchmark. The final model had an apparent AUC of 0.779 versus 0.648 for the routine-information model; the optimism-corrected AUCs were 0.751 and 0.608, respectively. The final model also had a lower Brier score and a calibration slope closer to 1, indicating advantages beyond discrimination (38, 48). Because the final model combines psychological and physical fitness dimensions, the comparison cannot isolate the independent contribution of either dimension. The nomogram is not intended to replace direct PSQI administration and would offer no clear advantage when the PSQI can be readily collected. Its potential role is opportunistic triage in community healthy-aging, obesity-management, or functional-assessment programs in which PHQ-9, GAD-7, and fitness measures are already obtained but sleep screening is not routinely embedded. In such settings, existing multidimensional data could be used to identify individuals for subsequent PSQI administration or clinical sleep assessment without adding a new first-stage assessment; this proposed use requires external validation and implementation evaluation. In the development sample, the final model showed greater theoretical net benefit across threshold probabilities of 13–80%. This range should not be interpreted as a community screening or intervention cutoff because DCA depends on the sample and assumed decision weights (49–51). Until independent external validation and a specific use context are established, the nomogram should be regarded as a research tool for community risk stratification rather than a basis for individual clinical decisions (52).

From an aging and public health perspective, the model may be most relevant in community programs where psychological screening and functional fitness assessments are already conducted. Integrating these measures may help prioritize individuals for more comprehensive sleep assessment without requiring laboratory-based testing. Implementation would nevertheless require consideration of staff training, assessment burden, referral pathways, and access to appropriate sleep services. Because the model was developed at a single center and has not undergone external validation, it should not yet be used to determine individual interventions or public health policy.

## 5 Strengths and limitations

This study has several strengths. It focuses on middle-aged and older adults with abdominal obesity, a metabolically relevant yet understudied population with a substantial burden of poor sleep quality, and integrates psychological and objective physical fitness measures within one risk-identification framework. Candidate predictors balanced evidence, clinical plausibility, community feasibility, and measurement burden. Repeated cross-validation and bootstrap resampling assessed selection stability, while a separate bootstrap procedure quantified optimism in the fixed seven-predictor model for discrimination, calibration, and prediction error; DCA assessed theoretical net benefit in the development sample. Comparison with a routine-information model in the same sample further quantified the added performance of the multidimensional model.

This study also has limitations. First, the retrospective cross-sectional design is susceptible to selection and information bias and precludes inferences regarding temporality, causality, or future sleep outcomes. Second, only internal validation was performed. Bootstrap optimism correction re-estimated coefficients after the seven-predictor structure had been fixed and did not repeat the full selection process. Although a separate bootstrap analysis assessed LASSO selection stability, optimism from the overall development process cannot be excluded; independent external validation is required before application. Third, participants came from one Beijing community, and the sample was relatively small. A *post hoc* Riley-framework assessment indicated that the available sample did not meet all conservative sample-size criteria; consequently, coefficient estimates and model performance may be less stable despite resampling-based internal validation. Generalizability to other regions, sex and age distributions, and other populations with abdominal obesity remains uncertain. Fourth, the PSQI measured self-reported sleep quality and was not supplemented by actigraphy or polysomnography. PHQ-9 Item 3 assesses sleep disturbance and overlaps conceptually with the PSQI. Because item-level PHQ-9 data were unavailable, a sensitivity analysis excluding this item could not be performed (23); consequently, the PHQ-9 association, overall discrimination, and apparent incremental performance may be overestimated. Finally, habitual snoring, shift-work status, chronic pain, menopausal status, and detailed use of medications other than hypnotics were unavailable and may contain additional predictive information. Abdominal obesity was defined by waist circumference, without direct measures of visceral adiposity or endocrine–metabolic biomarkers; therefore, the proposed mechanisms could not be evaluated in this study. Larger multicenter prospective studies should evaluate external calibration, discrimination, and potential use with objective sleep measures and more comprehensive health information.

## 6 Conclusion

This study developed and internally validated a nomogram that integrates psychological and physical fitness indicators to estimate the probability of current poor sleep quality among community-dwelling middle-aged and older adults with abdominal obesity. The seven-predictor model showed moderate discrimination and acceptable internal performance and outperformed a routine-information model. The combined psychological and physical-function information may support targeted sleep assessment in community healthy-aging programs where these measures are already available. External validation in independent and geographically diverse populations is required before implementation in public health practice.

## Data Availability

The deidentified data supporting the conclusions of this article are available from the corresponding author upon reasonable request, subject to applicable ethical and data-protection requirements.
