# Supplementary material for: Community-based identification of poor sleep quality in middle-aged and older adults with abdominal obesity using psychological and physical fitness indicators
Source: Front Public Health. 2026 Oct 21;14:1964356. doi: 10.3389/fpubh.2026.1964356 (PMC13730819; doi:10.3389/fpubh.2026.1964356)
Supplement: Supplementary file 1 [file Data_sheet_1.docx]

Supplementary Material

# 1 Supplementary Data

This supplement reports prespecified details of candidate-predictor coding, LASSO tuning and selection stability, final-model diagnostics, fixed-structure internal validation, the routine-information benchmark model, and decision-curve results. All analyses used the same 212 participants included in the final analysis (117 with PSQI > 5 and 95 with PSQI ≤ 5). No participant-level data are presented.

## **Supplementary Methods S1. Detailed model-development and internal-validation procedures**

Ten candidate predictors were defined before model fitting: age, sex, Patient Health Questionnaire-9 (PHQ-9), Generalized Anxiety Disorder-7 (GAD-7), Mini-Mental State Examination (MMSE), grip strength, 30-s chair stand, chair sit-and-reach, 2-min step test, and 8-Foot Up-and-Go test (8UG). Candidate definitions and scaling are summarized in Supplementary Table S1. The binary outcome was current poor sleep quality, defined as Pittsburgh Sleep Quality Index (PSQI) > 5.

Variable selection used least absolute shrinkage and selection operator (LASSO) logistic regression with α = 1. Continuous predictors were standardized within the LASSO procedure. We performed 100 repetitions of stratified 10-fold cross-validation; the penalty minimizing the mean cross-validated binomial deviance (λ_min) was the primary rule, with the one-standard-error penalty (λ_1SE) examined as a secondary rule. Selection stability was then assessed in 1000 bootstrap resamples, repeating LASSO tuning within every resample. Selection frequencies and coefficient-direction consistency are reported in Supplementary Table S2 and Supplementary Figure S1.

The seven predictors with nonzero coefficients at λ_min were entered simultaneously into an unpenalized multivariable logistic regression model. Variance inflation factors (VIFs), the design-matrix condition number, convergence, and bootstrap coefficient stability were examined (Supplementary Table S3). For internal validation of the fixed seven-predictor model, 1000 bootstrap resamples were used to re-estimate coefficients without repeating variable selection. Optimism-corrected discrimination, calibration, and overall prediction error are shown in Supplementary Table S4.

A routine-information benchmark model included age, sex, number of disease categories, current drinking, and exercise level (Supplementary Table S5). Because the benchmark and final models were non-nested, their correlated apparent AUCs were compared using a paired DeLong test; other between-model performance differences were estimated by 1000 paired bootstrap resamples. Decision-curve analysis quantified theoretical net benefit within the development sample across threshold probabilities from 5% to 80% (Supplementary Table S6). These thresholds should not be interpreted as externally validated intervention cutoffs.

# 2 Supplementary Figures and Tables

## **Supplementary Figures**

**Supplementary Figure S1.** Bootstrap selection frequency of the 10 candidate predictors.

Bars show the percentage of 1000 bootstrap resamples in which each predictor was selected after LASSO tuning was repeated within each resample. The dashed line is a 70% descriptive reference for selection stability and did not determine the final seven-predictor model. GAD-7, Generalized Anxiety Disorder-7; MMSE, Mini-Mental State Examination; PHQ-9, Patient Health Questionnaire-9.

**Supplementary Figure S2.** Spearman correlations among the 10 candidate predictors.

The lower-triangular matrix presents pairwise Spearman correlation coefficients among the 10 candidate predictors in the 212 participants included in model development. Cell values represent Spearman’s ρ; blue and red indicate negative and positive correlations, respectively, with color intensity reflecting correlation magnitude. Sex was coded as 0 for male and 1 for female. Black borders were prespecified to identify correlations with |ρ| ≥ 0.70; no off-diagonal predictor pair met this threshold. GAD-7, Generalized Anxiety Disorder-7; MMSE, Mini-Mental State Examination; PHQ-9, Patient Health Questionnaire-9.

**Supplementary Tables**

**Supplementary Table S1.** Definitions, coding, and completeness of the candidate predictors.

| **Predictor** | **Type** | **Definition/unit** | **Model scale** | **Available n/N** | **Selected at λ_min** |
| --- | --- | --- | --- | --- | --- |
| Age | Continuous | Years | Per 5 years | 212/212 | Yes |
| Sex | Categorical | Male/female | Indicator variable | 212/212 | No |
| PHQ-9 | Continuous | 0–27 points; higher scores indicate more depressive symptoms | Per 1 point | 212/212 | Yes |
| GAD-7 | Continuous | 0–21 points; higher scores indicate more anxiety symptoms | Per 1 point | 212/212 | Yes |
| MMSE | Continuous | Total score; higher scores indicate better cognition | Per 1 point | 212/212 | No |
| Grip strength | Continuous | Maximum grip strength (kg) | Per 5 kg | 212/212 | Yes |
| 30-s chair stand | Continuous | Completed repetitions in 30 s | Per 1 repetition | 212/212 | Yes |
| Chair sit-and-reach | Continuous | Best distance (cm); higher values indicate greater flexibility | Per 5 cm | 212/212 | Yes |
| 2-min step test | Continuous | Completed steps in 2 min | Per 10 steps | 212/212 | Yes |
| 8-Foot Up-and-Go test | Continuous | Shortest completion time (s) | Per 1 s | 212/212 | No |

The outcome was current poor sleep quality, defined as PSQI > 5. GAD-7, Generalized Anxiety Disorder-7; LASSO, least absolute shrinkage and selection operator; MMSE, Mini-Mental State Examination; PHQ-9, Patient Health Questionnaire-9; PSQI, Pittsburgh Sleep Quality Index.

**Supplementary Table S2.** LASSO tuning results and bootstrap variable-selection stability.

**Panel A. Penalty parameters and selected variables**

| **Selection rule** | **λ** | **log(λ)** | **No. selected** | **Selected predictors** |
| --- | --- | --- | --- | --- |
| λₘᵢₙ | 0.02220 | −3.8076 | 7 | Age; PHQ-9; GAD-7; grip strength; 30-s chair stand; chair sit-and-reach; 2-min step test |
| λ₁ₛₑ | 0.02674 | −3.6215 | 7 | Age; PHQ-9; GAD-7; grip strength; 30-s chair stand; chair sit-and-reach; 2-min step test |

**Panel B. Selection frequency across 1000 bootstrap resamples**

| **Predictor** | **Selection frequency (%)** | **Direction consistency among selected resamples (%)** |
| --- | --- | --- |
| PHQ-9 | 100.0 | 100.0 |
| GAD-7 | 83.8 | 99.9 |
| Chair sit-and-reach | 73.2 | 98.4 |
| Age | 72.8 | 96.6 |
| Grip strength | 68.3 | 99.0 |
| 30-s chair stand | 62.3 | 96.0 |
| 2-min step test | 59.9 | 92.5 |
| MMSE | 50.3 | 83.9 |
| 8-Foot Up-and-Go test | 36.7 | 59.9 |
| Sex | 34.6 | Not applicable |

The mean number of selected predictors was 6.419 (SD 2.033; median 6, interquartile range 5–8; range 1–10). All 1000 bootstrap resamples were successfully analyzed. Direction consistency is the percentage of selected resamples in which the coefficient had the same sign as in the original-sample LASSO model. Sex was represented by a categorical design-matrix term; a single direction-consistency percentage was therefore not reported. λ_min, penalty with minimum mean cross-validated deviance; λ_1SE, largest penalty within one standard error of the minimum.

**Supplementary Table S3.** Final-model diagnostics and bootstrap coefficient stability.

**Panel A. Model diagnostics**

| **Diagnostic** | **Value** |
| --- | --- |
| Maximum VIF | 1.298 |
| VIF range | 1.103–1.298 |
| Design-matrix condition number | 2.408 |
| Model convergence | Yes (5 Fisher-scoring iterations) |
| Null deviance | 291.607 |
| Residual deviance | 235.924 |
| AIC | 251.924 |
| Fitted probability range | 0.123–0.999 |
| Successful coefficient-stability resamples | 1000/1000 |

**Panel B. Coefficient stability across 1000 bootstrap resamples**

| **Predictor** | **Original β** | **Bootstrap mean β** | **Bootstrap SD** | **Bootstrap 95% interval** | **Direction consistency (%)** |
| --- | --- | --- | --- | --- | --- |
| Age, per 5 years | 0.115 | 0.114 | 0.140 | −0.153 to 0.393 | 80.6 |
| PHQ-9, per 1 point | 0.318 | 0.331 | 0.082 | 0.183 to 0.490 | 100.0 |
| GAD-7, per 1 point | 0.110 | 0.120 | 0.079 | −0.023 to 0.274 | 95.5 |
| Grip strength, per 5 kg | −0.114 | −0.116 | 0.107 | −0.320 to 0.093 | 86.4 |
| 30-s chair stand, per repetition | −0.023 | −0.026 | 0.043 | −0.112 to 0.055 | 71.7 |
| Chair sit-and-reach, per 5 cm | −0.082 | −0.091 | 0.077 | −0.251 to 0.052 | 88.1 |
| 2-min step test, per 10 steps | −0.063 | −0.064 | 0.118 | −0.303 to 0.161 | 69.9 |

In every bootstrap resample, the fixed seven-predictor structure was refitted without repeating LASSO selection. Direction consistency is the percentage of bootstrap coefficients with the same sign as the original estimate. AIC, Akaike information criterion; GAD-7, Generalized Anxiety Disorder-7; PHQ-9, Patient Health Questionnaire-9; VIF, variance inflation factor.

**Supplementary Table S4.** Bootstrap optimism correction for the fixed seven-predictor model.

| **Performance measure** | **Apparent** | **Mean bootstrap training** | **Mean original-sample test** | **Mean optimism** | **Optimism-corrected** | **Corrected 95% interval** | **Successful resamples** |
| --- | --- | --- | --- | --- | --- | --- | --- |
| AUC/C-index | 0.779 | 0.792 | 0.764 | 0.028 | 0.751 | 0.693–0.812 | 1000 |
| Calibration intercept | 0.000 | 0.000 | 0.002 | −0.002 | 0.002 | −0.327–0.340 | 1000 |
| Calibration slope | 1.000 | 1.000 | 0.858 | 0.142 | 0.858 | 0.606–1.154 | 1000 |
| Brier score | 0.190 | 0.183 | 0.198 | −0.015 | 0.206 | 0.181–0.238 | 1000 |

The seven-predictor structure was held fixed. Coefficients were re-estimated in each bootstrap sample and performance was evaluated in both the bootstrap sample and the original sample. The optimism-corrected value equals apparent performance minus mean optimism. AUC, area under the receiver operating characteristic curve.

**Supplementary Table S5.** Routine-information benchmark model: coding and coefficient estimates.

**Panel A. Predictor coding**

| **Predictor** | **Coding** |
| --- | --- |
| Age | Continuous; interpreted per 5-year increase |
| Sex | Female versus male |
| Number of disease categories | 0 (reference), 1, or ≥2 |
| Drinking | Current drinking versus not currently drinking |
| Exercise level | 1 = lowest, 2 = intermediate, 3 = highest; modeled per one-level increase |

**Panel B. Multivariable logistic regression estimates**

| **Term** | **β** | **SE** | **OR** | **95% CI for OR** | **P value** |
| --- | --- | --- | --- | --- | --- |
| Intercept | −4.366 | 1.680 | 0.013 | 0.000–0.342 | 0.009 |
| Age, per 5 years | 0.269 | 0.108 | 1.308 | 1.059–1.616 | 0.013 |
| Female versus male | 0.550 | 0.369 | 1.733 | 0.842–3.569 | 0.135 |
| 1 disease versus 0 | 1.559 | 0.818 | 4.752 | 0.957–23.596 | 0.057 |
| ≥2 diseases versus 0 | 1.178 | 0.729 | 3.247 | 0.778–13.557 | 0.106 |
| Current drinking | 0.600 | 0.365 | 1.822 | 0.891–3.725 | 0.100 |
| Exercise, per one level | −0.338 | 0.184 | 0.713 | 0.497–1.024 | 0.067 |

CI, confidence interval; OR, odds ratio; SE, standard error. The benchmark model was used for performance comparison and was not intended as a standalone externally validated clinical model.

**Supplementary Table S6.** Decision-curve results at representative threshold probabilities.

**Panel A. Net benefit**

| **Threshold** | **Final model** | **Routine model** | **Treat all** | **Treat none** | **ΔNB: final − routine** | **ΔNB: final − best default** |
| --- | --- | --- | --- | --- | --- | --- |
| 5% | 0.528 | 0.528 | 0.528 | 0.000 | 0.000 | 0.000 |
| 10% | 0.502 | 0.502 | 0.502 | 0.000 | 0.000 | 0.000 |
| 20% | 0.448 | 0.441 | 0.440 | 0.000 | 0.007 | 0.008 |
| 30% | 0.390 | 0.367 | 0.360 | 0.000 | 0.023 | 0.030 |
| 40% | 0.318 | 0.247 | 0.253 | 0.000 | 0.071 | 0.064 |
| 50% | 0.236 | 0.146 | 0.104 | 0.000 | 0.090 | 0.132 |
| 60% | 0.170 | 0.111 | -0.120 | 0.000 | 0.059 | 0.170 |
| 70% | 0.187 | 0.053 | -0.494 | 0.000 | 0.134 | 0.187 |
| 80% | 0.090 | 0.028 | -1.241 | 0.000 | 0.061 | 0.090 |

**Panel B. Interventions avoided per 100 participants**

| **Threshold** | **Final model versus treat all** | **Final model versus routine model** |
| --- | --- | --- |
| 5% | 0.0 | 0.0 |
| 10% | 0.0 | 0.0 |
| 20% | 3.3 | 2.8 |
| 30% | 7.1 | 5.3 |
| 40% | 9.7 | 10.6 |
| 50% | 13.2 | 9.0 |
| 60% | 19.3 | 3.9 |
| 70% | 29.2 | 5.7 |
| 80% | 33.3 | 1.5 |

Net benefit values are development-sample estimates. “Best default” is the greater net benefit of treat all and treat none at each threshold. The reported thresholds are illustrative and must not be interpreted as externally validated intervention cutoffs. ΔNB, difference in net benefit.
